# Learning nonwords: the Hebb repetition effect as a model of word learning

**DOI:** 10.1080/09658211.2017.1416639

**Published:** 2018-01-03

**Authors:** Dennis Norris, Michael P. A. Page, Jane Hall

**Affiliations:** a MRC Cognition and Brain Sciences Unit, Cambridge, UK; b Department of Psychology, University of Hertfordshire, Hatfield, UK

**Keywords:** Short-term memory, Hebb task, word learning, long-term memory, learning

## Abstract

Page and Norris [(2008). Is there a common mechanism underlying word-form learning and the Hebb repetition effect? Experimental data and a modelling framework. In A. Thorn & M. P. A. Page (Eds.), *Interactions between short-term and long-term memory in the verbal domain*; (2009). A model linking immediate serial recall, the Hebb repetition effect and the learning of phonological word forms. *Philosophical Transactions of the Royal Society B: Biological Sciences*, *364*(1536), 3737–3753. doi:10.1098/rstb.2009.0173] have suggested that the Hebb [(1961). Distinctive features of learning in the higher animal. In J. F. Delafresnaye (Ed.), *Brain mechanisms and learning* (pp. 37–46). Oxford: Blackwell] repetition paradigm can be considered as a laboratory analogue of word learning. In Hebb learning experiments, the lists of items to be learned are presented as discrete sequences. In contrast, novel words are, by definition, always heard as a single coarticulated whole. Might this undermine the claim that Hebb learning can shed light on word learning? Here we report an experiment comparing learning sequences of isolated syllables with learning the same sequences spoken as a single coarticulated nonword. The pattern of learning was similar in the two cases, suggesting that the Hebb repetition paradigm can indeed provide valuable insights into the way novel word forms are learned.

In order to learn the phonological form of a new word, infants and adult learners must be able to hold a representation of that word in some form of temporary memory so that the word as a whole can be committed to long-term memory. Baddeley, Gathercole, and Papagno (1998) marshalled extensive evidence in support of the claim that this temporary storage is provided by the phonological store component of the working memory model (Baddeley, 1986; Baddeley & Hitch, 1974). Once a child has learned the basic repertoire of speech sounds in their language, the process of learning the form of a new word becomes one of learning the order in which those sounds appear. The primary role of the phonological store in learning new words is therefore to retain the order of those sounds.

The processing requirements of word learning have an interesting parallel with the laboratory task introduced by Hebb (1961). Hebb had participants perform immediate serial recall of lists of digits where, unannounced to the participants, every third list was repeated. Performance improved over repetitions. Although the task is simply to recall the lists, repeated presentations of the lists lead to long-term learning. The Hebb paradigm, therefore, seems to involve the two main components of learning new words; holding a sequence of speech sounds in short-term memory, and transferring that sequence to long-term memory. We have suggested that vocabulary acquisition and performance in the Hebb paradigm are both subserved by the same processes, and that Hebb learning can, therefore, be taken as a laboratory model of word form learning (Page & Norris, 2008, 2009). This certainly seems to be the most parsimonious assessment of the relation between the two tasks. If learning word forms and learning sequences in the Hebb paradigm were completely unrelated this would imply that there were two quite distinct mechanisms for learning about sequences of phonological information. The simplest view is clearly that there is a single mechanism for learning phonological sequences that supports both word learning and Hebb learning. This receives support from a study by Mosse and Jarrold (2008) who found a correlation between learning in a Hebb task and learning to associate nonwords with pictures in children of about six years of age. Additional evidence for the strong relationship between the Hebb effect and word learning comes from a study in Dutch by Szmalec, Page, and Duyck (2012) who exposed participants to visually presented lists of nine consonant–vowel syllables. Some three-syllable sequences within those lists were repeated on every third trial (e.g., *la-va-bu*). The repeated sequences were nonwords designed to be orthographic neighbours of existing Dutch base words (*lavabo*). In a subsequent auditory lexical decision task, those repeated nonwords behaved as if they had become lexicalised and functioned as competitors to the base words. The Hebb repetition paradigm, therefore, holds promise as a tractable experimental model of the processes involved in learning new words.

The case for viewing the Hebb effect as a useful model of word learning has been presented in detail by Page and Norris (2008, 2009). They pointed out that if the Hebb effect is to be used as a model of word learning, the two must share several critical properties. First, in the same way that it must be possible to learn many words at once, it should also be possible to learn more than one Hebb list at a time (Kalm, Davis, & Norris, 2013; Kalm & Norris, 2015; Page, Cumming, Norris, McNeil, & Hitch, 2013). Second, learning must be long-lasting. Page et al. showed that memory for lists presented in a Hebb paradigm could persist for at least three months. Finally, learning must be possible even when repetitions are widely spaced. In the standard Hebb paradigm lists repeat every three trials. Page et al. found significant Hebb learning even when the critical lists repeated only every 12 trials. Longer spacings could not be investigated in a single experimental session. Note that few studies of Hebb learning have used spoken rather than written Hebb lists. Smalle et al. (2016) reported three Hebb learning experiments using spoken syllables. In their third experiment, they compared Hebb learning of lists of isolated syllables with lists where the syllables were grouped into pairs by inserting 1000 ms pauses between pairs of CV syllables. They argued that the grouping manipulation made the syllables function as larger chunks. They found that when the Hebb and filler syllables were drawn from the same pool, grouping increased the size of the Hebb effect. However, whereas their experiment takes isolated syllables and inserts extra pauses, what we would like to do it to compare isolated syllables with naturally coarticulated nonwords. Indeed, while these studies have shown that learning in the Hebb paradigm shares properties with learning phonological word forms, we have yet to show that novel word forms, as opposed to sequences of digits, words, letters or syllables, can be learned with a standard Hebb procedure.

Of course, in the broadest sense, we already know that children and adults can learn novel sequences of phonemes; this is exactly what word learning involves. The question here is whether learning nonwords shows the same pattern of learning as does learning sequences of items. To this end, we compared learning sequences of discrete nonsense syllables with learning longer nonwords consisting of the same sequences of syllables spoken as a single item. One important difference between a sequence of discrete syllables and a fluently spoken longer nonword is that the syllables in the latter will necessarily be coarticulated. Archibald and Gathercole (2007a, 2007b; Archibald, Gathercole, & Joanisse, 2009) showed that, as might be expected, naturally spoken nonwords are easier to recall than the same sequence of syllables spoken in isolation. Nevertheless, in line with Gupta (2005), Archibald and Gathercole (2009) found that nonword repetition showed the same pattern of primacy and recency effects seen in serial recall. These findings are promising as they show that nonword repetition is qualitatively similar to immediate serial recall of isolated syllables. However, the outstanding question is whether nonword learning shows the same characteristics as list learning in the Hebb paradigm. Here we examine this directly.

## Method

### Participants

There were 26 participants selected from the MRC Cognition and Brain Sciences Unit (CBU) volunteer panel, consisting of 17 females and 9 males, with a mean age of 19.5 years (range 16–24). All were native speakers of English, and each received a small honorarium for their participation.

In order to maximise the chances of obtaining a reliable Hebb effect, list length was tailored to each subject by first determining the subject’s span for nonwords. The critical Hebb and control lists for each participant were then set to one syllable greater than their span. Nineteen participants had a span of seven, six a span of six, and one a span of five. As so few participants had spans below 7, we restricted our analysis to the 19 span-7 participants.

### Stimuli and design

The stimuli were constructed from a set of 202 CV and CVC syllables. In order to ensure that stimuli sounded natural when spoken as coarticulated nonwords some of the syllables were real words. For example, we used CV syllables with tense vowels such as keI and wi, and the letter names di and ji. If we count proper name and recent coinages (e.g., mԑ, lit), up to 40 of the syllables could be interpreted as words. Given that new words that one may need to learn can be composed of existing words, there was no need to limit ourselves to syllables that were not words. Each list in the main part of the experiment consisted of a sequence of between five and eight syllables. The final syllable was always a CVC and the remainder were always CVs. This was done to make the stimuli sound more natural when pronounced as a single nonword. The fluent-nonword and isolated-syllable versions of the stimuli were recorded in a soundproof booth using a high-quality microphone. Stimuli were spoken at a normal speech rate by a female speaker of British English. They were edited and down-sampled to 22.05 kHz to create separate .wav files for each complete nonword and each syllable.

An additional 40 nonwords (10 each at syllable lengths 4, 5, 6 and 7) were recorded for the span test, as well as 4 practice trials for the 1-syllable-at-a time condition. All sequences were constructed so that they only contained transitions between the syllables that occurred in English words. Apart from the mode of presentation, the structure of the nonword and syllable blocks was the same. There were two different sets of stimuli, each of which was used to construct two running orders differing only in terms of the presentation mode of each half of the list. All participants heard one block of syllable trials and one block of nonword trials.

There were two repeated Hebb lists in each half of the experiment. One Hebb list first appeared on trial three and the other on trial five. In effect, the lists had a structure which repeated every block of six trials: filler, filler, Hebb A, filler, Hebb B, control filler, where the control filler was the matched control list for comparison with the Hebb lists. Each filler and control list appeared only once. The control filler was the same length as the Hebb lists but the remaining fillers were five, six or seven syllables long (in the case of the span-7 condition). Each Hebb list was presented eight times in total.

The materials consisted of 2 blocks, each containing 48 experimental trials. Each participant performed one block where the syllables were presented as a single fluently spoken nonword and one block where the syllables were presented with clear pauses in between, at a rate of 850 ms per syllable. The ordering of the two blocks was counterbalanced across participants.

In the span-7 condition, each subject heard 96 trials, which included repetitions of the 4 Hebb lists (2 in each condition). Collapsing over repetitions of the Hebb lists there were 448 syllables in total. These syllables were constructed from the base set of 202 different syllables which occurred between 1 and 9 times each, with approximately half of those syllables occurring only once. All filler lists appeared only once. In addition to the matched control fillers, there were also eight fillers of lengths 5, 6 and 7. This meant that participants could not be certain how many syllables there were in each list.

### Procedure

Each participant’s span was measured using the same presentation procedure as for the nonword block of the main experiment. Stimulus presentation was controlled by DMDX (Forster & Forster, 2003) and stimuli were presented over Sennheiser headphones.

Subjects initiated each trial by pressing the spacebar. The word “READY” was displayed on the computer screen in front of the participant for 500 ms before presentation of the spoken list. In the discrete syllable condition, one syllable was presented every 850 ms. Immediately after the end of the list the word “RECALL” appeared on the screen for 3 sec. Participants were asked to repeat what they had heard as clearly and accurately as possible. Spoken responses were recorded so that they could be transcribed and scored off-line. The Span Test acted as practice trials for the complete nonword condition, whereas there were four practice trials before the one-syllable-at-a-time condition.

Span was measured by presenting up to 10 trials at each list length beginning with lists of length 4. Participants were asked to repeat what they heard as clearly and accurately as possible. If or when the participant got four trials in a row correct the length was increased by one syllable. If, on the list length where they failed to get four in a row correct, they nevertheless got 50% correct, this length was deemed to be their span.

## Results

**Figure 1.**
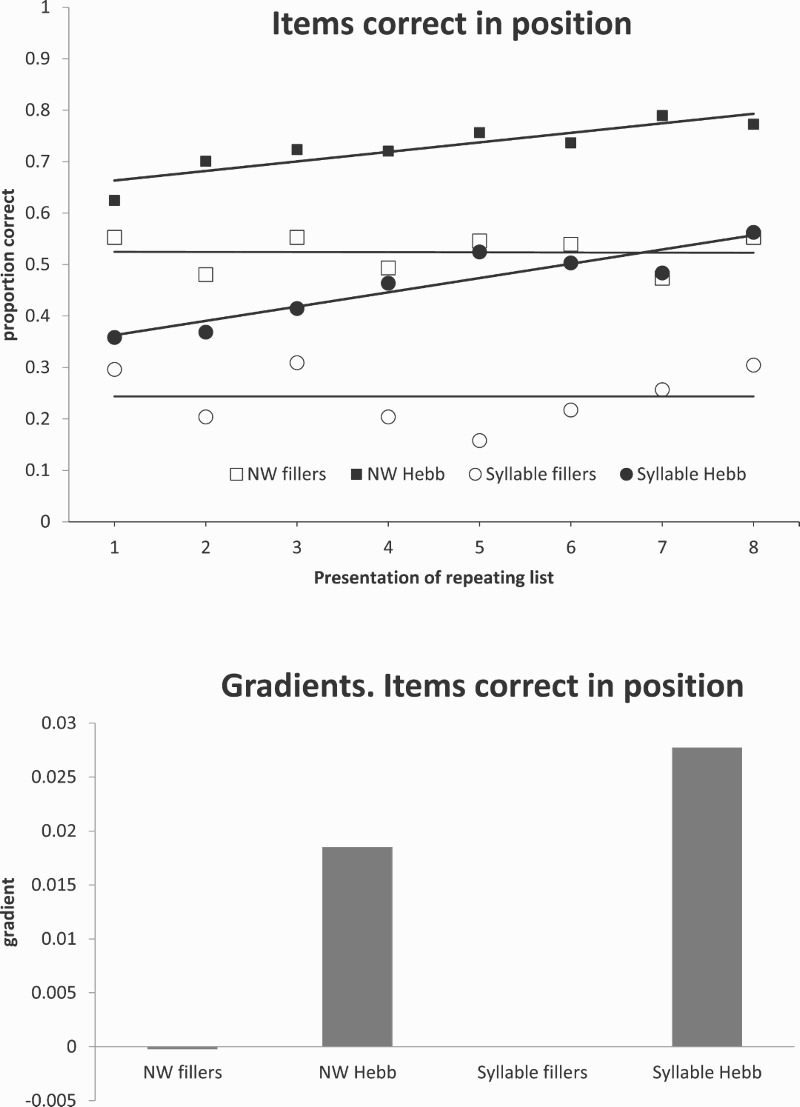
(a) Items correct in position. (b) Gradients – items correct in position.

Figure 1(a) plots recall accuracy for Hebb and control lists scored in terms of syllables recalled in their correct position. Gradients of change in performance across blocks are shown in Figure 1(b). The results of this experiment are very straightforward. There is a significant Hebb effect in both the isolated syllable and the nonword conditions, and this holds regardless of whether recall is scored for items in position, or for items recalled regardless of position. There are no significant interactions with presentation style (*F*s < 1). In line with Archibald and Gathercole’s results, nonword recall is generally better than syllable recall.

In the analysis of a Hebb paradigm, the crucial dependent variable is the slope of recall accuracy over repetitions. The primary analyses are performed on the slopes of least-squares regression lines representing change in performance over blocks. Hebb trials should show a positive slope, and this slope should be greater than for filler trials. Even filler trials might possibly have a positive slope if there is a practice effect whereby overall performance improves throughout the experiment. There was a positive gradient for both syllable (*t*(18) = 6.77, *p* < .001) and nonword Hebb lists (*t*(18) = 3.70, *p* < .001) and no hint of a slope for filler lists (nonwords: *t*(18) = 0.27; syllables: *t*(18) = 0.36). In addition, there was a significant difference between the slopes of Hebb and filler lists (*F*(1,18) = 28.4, *p* < .001), but no main effect of presentation style (discrete syllable versus nonword) and no interaction between style and list type (*F*s < 1). The results for item only-scoring showed the same pattern (*F*(1,18) = 27.8, *p* < .001). In contrast, there was no significant slope for any condition when scored in terms of items in position as a proportion of items correctly recalled (all *p*s > .15).

Although not reported here, we also analysed recall of consonants and vowels separately. Regardless of scoring method, there were significant Hebb effects for both, with the only additional finding being that vowels were recalled better than consonants. This is most likely because there are more consonants than vowels in the language.

Although the critical analyses are those using gradients, analyses of recall accuracy are also illuminating. In all analyses, nonword recall was better than syllable recall (correct in position: *F*(1,18) = 160.985, *p* < .001; item correct: *F*(1,18)= 92.203, *p* < .001). This was so even in the case of position scoring as a proportion of items recalled (*F*(1,18) = 73.825, *p* < .001). That is, even when a syllable was correctly recalled, it was less likely to be recalled in the correct position than was the corresponding syllable in a nonword.

## Discussion

The items in a conventional Hebb paradigm are discrete sequences of words or syllables. However, words are spoken as smoothly coarticulated wholes. Here we have shown that both show the same pattern of improvement over repeated presentations in a Hebb task. This is consistent with Page and Norris’s (2008, 2009) suggestion that the Hebb paradigm can be considered to be a laboratory analogue of word learning.

**Figure 2.**
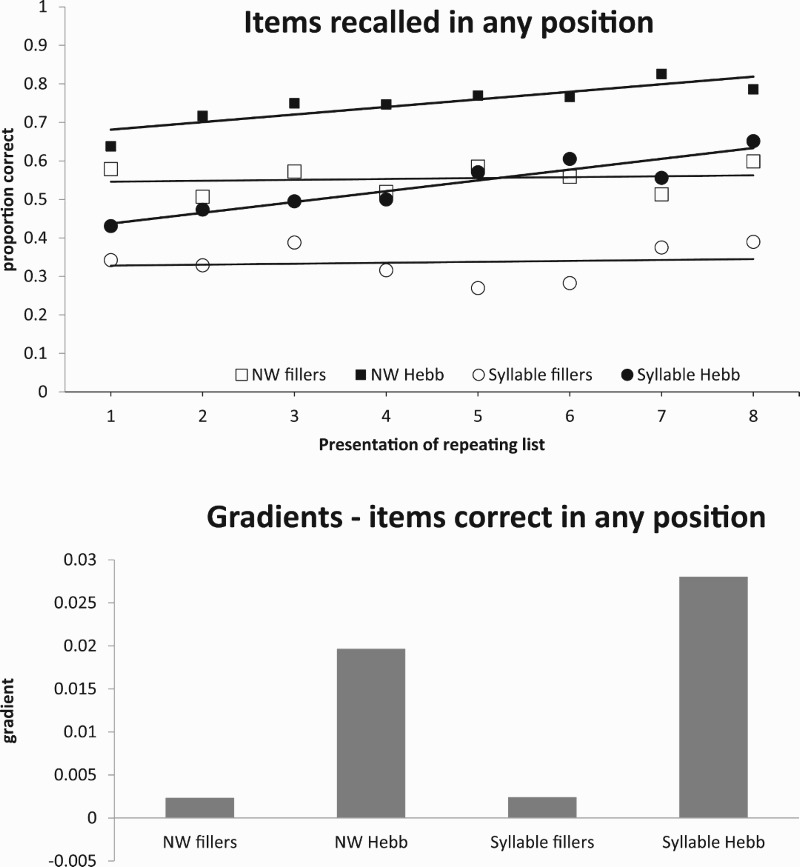
(a) Items recalled in any position. (b) Gradients – items correct in any position.

**Figure 3.**
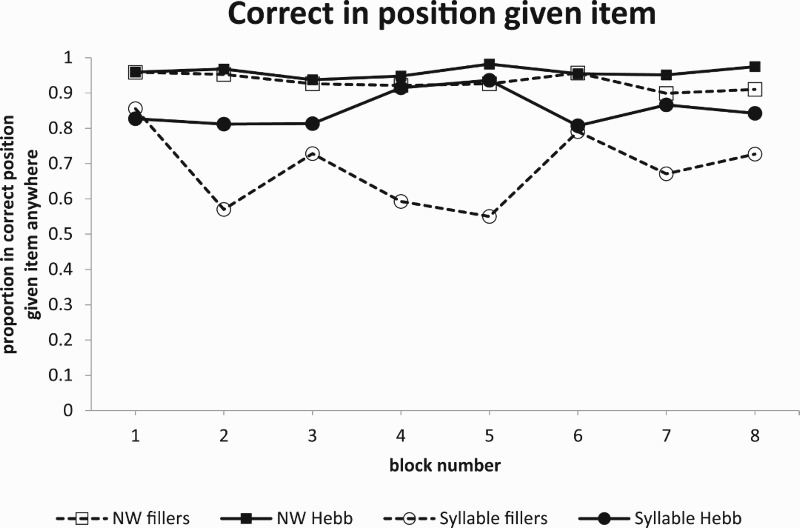
Correct in position given item.

In a Hebb paradigm using familiar stimuli, participants need to learn two things – the items, and their order. Experiments using the Hebb paradigm usually focus on learning order, and therefore Hebb and filler items are drawn from the same set. However, participants must also learn the set of items used in the experiment. With unfamiliar stimuli such as the syllables used here, participants also have to learn the individual syllables themselves. Could it be that the improvement over repetitions is simply a matter of learning the syllables rather than their order? This could apply equally to both the syllable and the nonword conditions. Figure 2 shows the learning curves scored for syllable correct, regardless of position. These are very similar to the item-in-position scores in that they also show a Hebb effect. This might seem to support the idea that subjects are simply learning the identity of the syllables, and not their order. However, Figure 3 shows the proportion of items recalled in the correct position given that the item was recalled anywhere in the list. Here performance is constant over repetitions. In other words, when subjects recall an item, they almost always recall it in the correct position. This could, for example, occur if subjects formed a frame in which items could be positioned once they were learned. Such a frame is likely to be much more constraining for nonwords where the extra coarticulatory and prosodic information would be expected to provide additional cues as to the position of items in the sequence. This might explain why recall in position as a proportion of items recalled is much worse for discrete syllable than for the nonword condition. Importantly though, this analysis demonstrates that subjects are not just learning about items because, once learned, the items generally appear in the correct order.

## Conclusions

The present results add to the body of data showing close parallels between learning of discrete sequences of items in the Hebb paradigm, and learning novel word forms. The pattern of learning is the same regardless of whether syllables are presented in isolation or in the form of a single coarticulated nonword. The only notable difference between the two is that, as previously observed by Archibald and Gathercole, performance is better for coarticulated nonwords. This result supports the view that Hebb learning is indeed a viable model for phonological word form learning, and can continue to provide insights into the acquisition of spoken word forms.

## References

[CIT0001] Archibald L. M. D., & Gathercole S. E. (2007a). Nonword repetition and serial recall: Equivalent measures of verbal short-term memory?, 28(4), 587–606. doi: 10.1017/S0142716407070324

[CIT0002] Archibald L. M. D., & Gathercole S. E. (2007b). Nonword repetition in specific language impairment: More than a phonological short-term memory deficit. , 14(5), 919–924. doi: 10.3758/BF03194122 18087960

[CIT0003] Archibald L. M. D., Gathercole S. E., & Joanisse M. F. (2009). Multisyllabic nonwords: More than a string of syllables. , 125(3), 1712–1722. doi: 10.1121/1.3076200 19275328

[CIT0004] Baddeley A. D. (1986). . Oxford: Oxford University Press.

[CIT0005] Baddeley A., Gathercole S., & Papagno C. (1998). The phonological loop as a language learning device. , 105(1), 158–173. doi: 10.1037/0033-295X.105.1.158 9450375

[CIT0006] Baddeley A. D., & Hitch G. J. (1974). Working memory. In Bower G. H. (Ed.), (Vol. 8, pp. 47–89). New York: Academic Press.

[CIT0007] Forster K. I., & Forster J. C. (2003). DMDX: A Windows display program with millisecond accuracy. , 35, 116–124. doi: 10.3758/BF03195503 12723786

[CIT0008] Gupta P. (2005). Primacy and recency in nonword repetition. , 13(3–4), 318–324. doi: 10.1080/09658210344000350 15948616

[CIT0009] Hebb D. O. (1961). Distinctive features of learning in the higher animal. In Delafresnaye J. F. (Ed.), (pp. 37–46). Oxford: Blackwell.

[CIT0010] Kalm K., Davis M., & Norris D. (2013). Individual sequence representations in the medial temporal lobe. , 25(7), 1111–1121. doi:10.1002/hbm.21308 23448522

[CIT0011] Kalm K., & Norris D. (2015). Recall is not necessary for verbal sequence learning. . doi:10.3758/s13421-015-0544-0 PMC4722071 26289546

[CIT0012] Mosse E. K., & Jarrold C. (2008). Hebb learning, verbal short-term memory, and the acquisition of phonological forms in children. , 61(4), 505–514. doi: 10.1080/17470210701680779 18300182

[CIT0013] Page M. P. A., Cumming N., Norris D., McNeil A. M., & Hitch G. J. (2013). Repetition-spacing and item-overlap effects in the Hebb repetition task. , 69, 506–526. doi: 10.1016/j.jml.2013.07.001

[CIT0014] Page M. P. A., & Norris D. (2008). Is there a common mechanism underlying word-form learning and the Hebb repetition effect? Experimental data and a modelling framework. In Thorn A. & Page M. P. A. (Eds.), (pp. 136–155). Hove: Psychology Press.

[CIT0015] Page M. P. A., & Norris D. (2009). A model linking immediate serial recall, the Hebb repetition effect and the learning of phonological word forms. , 364(1536), 3737–3753. doi: 10.1098/rstb.2009.0173 PMC2846317 19933143

[CIT0016] Smalle E. H. M., Bogaerts L., Simonis M., Duyck W., Page M. P. A., Edwards M. G., & Szmalec A. (2016). Can chunk size differences explain developmental changes in lexical learning?, 6. doi: 10.3389/fpsyg.2015.01925 PMC4703851 26779065

[CIT0017] Szmalec A., Page M., & Duyck W. (2012). The development of long-term lexical representations through Hebb repetition learning. , 67(3), 342–354. doi: 10.1016/j.jml.2012.07.001

